# Molecular interpretation of the carbon nitride performance as a template for the transport of anti-cancer drug into the biological membrane

**DOI:** 10.1038/s41598-021-98597-2

**Published:** 2021-09-23

**Authors:** Ameneh Zaboli, Heidar Raissi, Farzaneh Farzad

**Affiliations:** Department of Chemistry, University of Birjand, Birjand, Iran

**Keywords:** Drug delivery, Computational biophysics

## Abstract

Evaluation of interaction mechanism between 2-dimensional (2D) nanomaterials and cell membranes is a critical issue in providing guidelines for biomedical applications. Recent progress in computer-aided molecular design tools, especially molecular dynamics (MD) simulation, afford a cost-effective approach to achieving this goal. In this work, based on this hypothesis, by utilizing theoretical methods including MD simulation and free energy calculations, a process is evaluated in which the Doxorubicin (DOX)-loaded onto carbon nitride (CN) nanosheet faced with bilayer membrane. It should be mentioned that to achieve an efficient CN-based drug delivery system (DDS), in the first place, the intermolecular interaction between the carrier and DOX is investigated. The obtained results show that the DOX prefers a parallel orientation with respect to the CN surface via the formation of π–π stacking and H-bond interactions. Furthermore, the adsorption energy value between the drug and the carrier is evaluated at about − 312 kJ/mol. Moreover, the investigation of the interaction between the CN-DOX complex and the membrane reveals that due to the presence of polar heads in the lipid bilayer, the contribution of electrostatic energy is higher than the van der Waals energy. The global minimum in free energy surface of the DDS is located between the head groups of the cell membrane. Overall, it can be concluded that the CN nanosheet is a suitable candidate for transfer and stabilize DOX on the membrane.

## Introduction

To date, nanotechnology has made significant advances in the development of drug delivery systems (DDSs)^1^. DDSs are used to improve drug bioavailability and reduce their side effects. Nanomaterials are used as carriers in the field of nanomedicine because the chemotherapy drugs can be loaded in their free internal volume^2^. Especially, 2-dimensional (2D) nanosheets are one of the most attractive nanocarriers which due to their distinctive properties, have significant potential in medical applications, drug delivery, and photodynamic therapy^2^.

It should be noted that contact between cell membranes and DDSs is an important issue in pharmacology, biomedicine, and biotechnology^3^. Phospholipids are the most abundant lipids in membrane structure that are self-assembled with their hydrophobic hydrocarbon tails and polar head groups^4^. On the other hand, physical damage to cell membranes by some of 2D nanocarriers causes cytotoxicity, which means some nanocarriers can be converted to novel antibiotics^5–7^. Also, it is shown that these carriers are successful in transporting some drugs that have difficulty crossing the membrane^8–10^.

Carbon nitride (CN) nanosheets due to their unique properties such as chemical inertness, thermal conductivity, structural stability, and good biological compatibility have attracted more attention among 2D nanomaterials^11–14^. Because of its specific structure, which is constructed by regular N-substituted graphite, this nanosheet exhibits many useful properties in various fields such as sensing, bioimaging, drug delivery, and photo-electrocatalyst^15–17^. For example, Liu et al. for the first time loaded a 2-phenylethynesulfonamide inhibitor onto a CN nanosheet as a multifunctional nanoplatform to enhance photodynamic therapy^8^. Their obtained results were shown that this carrier with strong blue fluorescence, good water dispersity, and proper drug loading capacity, can concurrently act as an imaging agent, a drug carrier, and a photosensitizer. Furthermore, in previous studies, it was suggested that the carbon nitride delivers the drug into the cell^18,19^. However, the molecular action mechanism is not clearly elucidated and there are few reports in which the interaction between the biological membrane and the CN was investigated. Comprehending the interaction between the biological membrane and CN is essential for the further use of CN for medical applications. Molecular Dynamics (MD) simulations provide the three-dimensional view from systems at the atomic level, which is difficult to obtain using experimental methods^20^. Previously, we using MD simulations to evaluate the loading of two anticancer drugs on the functional and pristine CN nanosheets^21^. The simulation results confirmed that this carrier can be used as a highly efficient DDS. However, the dynamics and structure of CN nanosheets in the presence of membranes have not yet been systematically investigated. Two different structures have been reported for the CN nanosheet (i.e., triazine and heptazine). Triazine CN built s-triazine units (ring of C3N3) with a periodic array of single-carbon vacancies. Heptazine CN includes the condensed tri-s-triazine (tri-ring of C6N7) subunits coupled with planar tertiary amino groups. In this work, triazine is selected as a model to be evaluated as a carrier for doxorubicin delivery. Pumera et al.^22^ reported that triazine and heptazine CNs have lower toxicity than graphene oxide nanosheet, which make them good candidates for biomedical applications. In another work, Lee and co-workers^18^ designed a core–shell nanoparticle based on zeolitic-imidazolate framework-8 and triazine. They found CN nanosheet led to a significant increase in the efficacy of DOX.

MD simulations conforming to the Boltzmann distribution sample the phase space, and high-energy sampling may not occur at achievable simulation times. Thus, in recent decades, to solve this problem the free energy calculations are employed. The free energy calculation methods are powerful tools in understanding many phenomena^23–25^.

In this study, MD simulations are performed to examine the loading of the doxorubicin (DOX) drug on the CN nanocarrier and explore the interactions of DDS with a lipid membrane. The well-tempered Metadynamics has been used to obtain free energy surface (FES) of diffusion prosses. Nevertheless, we hope that our study results can be useful for future studies in this field.

## Computational methods

MD simulation is performed to provide molecular insights into the ability of the CN nanosheet in drug transportation. Initially, the nanocarrier is positioned at the centre of an 8 × 8 × 8 nm^3^ box. Then, four molecules of DOX drug are placed approximately at an initial distance of 2 nm away from the CN surface. The initial structure of the CN is composed of 360 carbon, 469 nitrogen, and 36 hydrogen atoms, which are constructed using Gauss view software^26^. To determine the partial charges of CN sheet atoms, quantum mechanical calculations at M06–2X/6–31G** level^27^are performed by using the Gaussian 09 program^28^.

The structure of the DOX drug molecule is given from the PubChem database^29^. This drug has aromatic rings that are prone to the formation of π–π interaction with CN. The topology and force field parameters of the DOX molecule are taken from the Swiss Param server^30^.

**Figure 1:**
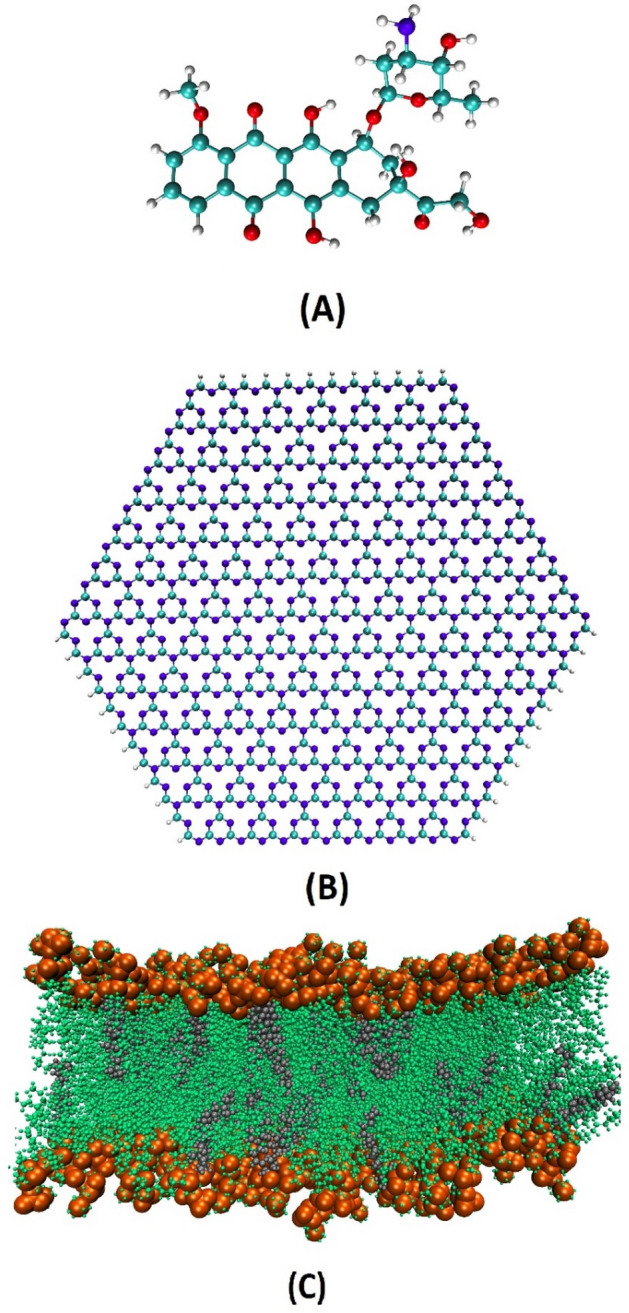
The structure of (**A**) Doxorubicin drug, (**B**) Carbon nitride nanosheet, (**C**) POPC bilayer, respectively.

Because the drug delivery performance of CN depends on its ability to diffuse to the cell membrane, the interaction of the CN-DOX complex with lipid bilayer is investigated. To examine this process the final orientation of the DOX molecule on the carrier is extracted from the loading system. In the following, this complex is located in parallel orientation above the membrane surface at a distance of ≈ 2 nm. The dimensions of this simulation box are considered 11.44 × 11.44 × 19 nm^3^. The selected membrane in this study contains 400 Palmitoyloleoylphosphatidylcholine (POPC) lipid and 100 cholesterol (CHL) about 20% of the total membrane, which is almost similar to the amount of CHL in the cancer cell membrane^31^. This membrane is fabricated using the protocol explained on the CHARMM-GUI website^32^ and then is equilibrated for 30 ns. In the present work to carry out all MD simulations, the GROMACS software package (version 2019.2)^33^ is used. The CHARMM36 force field^34^ is chosen to obtain the carrier and membrane parameters. Figure 1 demonstrates the chemical structure of the CN nanosheet, the DOX molecule, and the bilayer membrane.

To consider the solvent molecules in the simulation boxes, the water molecules using the TIP3P model are explicitly treated^35^. Na^+^ and Cl^−^ ions are added at a concentration of 0.15 to neutralize the system and mimic the biological environment. In addition, an energy minimization using the steepest descent algorithm is executed for each system. Then, two equilibration steps in NVT and NPT ensembles are run for 200 ps to temperature and pressure reach their constant values. In both simulation systems, the temperature at 310 K and pressure at 1 atm are kept constant using the Nose–Hoover thermostat^36^, and Parrinello-Rahman barostat^37^, respectively. MD simulations for the loading and the membrane systems under boundary conditions in three directions are run for 100 ns and 200 ns, respectively. Besides, the integration time step is set to 0.0015 fs. In these systems for maintaining all of the bonds at their equilibrium position, the LINCS algorithm is used. The van der Waals (vdW) interactions at a cut-off distance of 1.4 nm are evaluated, and to calculate Electrostatic (Ele) forces, Particle-mesh Ewald (PME) method is applied. In order to visualize the simulation results, the VMD software is employed.

### Free energy calculation

To accurately evaluate the diffusion process of the DDS into the membrane, the free energy calculation is performed. It has been proven that free energy calculation can be used as a powerful tool in the study of biological systems. Among the emerging techniques, metadynamics is an FES calculation method that is performed in this work using PLUMED (version 2.5.2)^38^.

In this method, the bias potential is smoothed in the long-time limit and the final FES can be appraised from the following statement^39^:

1 $${V}_{bias}\left(s,t\to \infty \right)=-\frac{\Delta T}{T+\Delta T}F\left(s\right)+C$$

Here, s, t, F(S), and ∆T are collective variables, time, free energy, and an input parameter with a temperature dimension that regulates the rate of FES exploration, respectively.

For these calculations, Gaussian hills are added with an initial height of 1.0 kJ mol^−1^ and a width of 0.25 nm every 500 ps. The FES profile is calculated as a function of the collective variable which is defined as the distance between the centre of mass (COM) of the POPC and the COM of the DDS.

## Results and discussion

In this section, a step-by-step approach is employed to evaluated the adsorption behaviour of DOX on CN nanocarrier and their interaction with the membrane cell using MD and well-tempered Metadynamics simulations. First, the loading of DOX drugs on the carrier is examined. Next, the penetration process of the DDS into the POPC is investigated.

### DOX adsorption on CN

**Figure 2:**
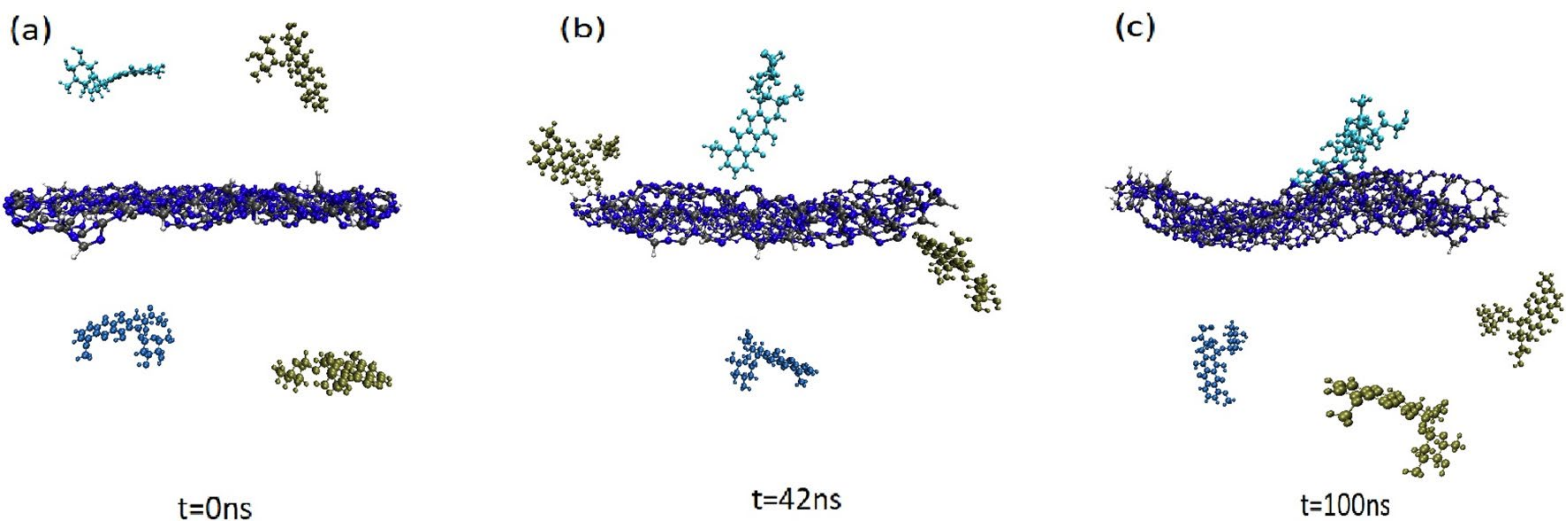
Representative snapshots corresponding to the interaction of DOX molecules with CN nanosheet at three different times: (**a**) 0 ns, (**b**) 42 ns, (**c**) 100 ns.

In this part, the interaction of DOX with CN is studied to explore the mechanisms involved in the adsorption process of the drug on the CN surface. Several snapshots at different time frames corresponding to this MD simulation are given in Fig. 2.

As it is clear from Fig. 2a, at the beginning of the simulation (0 ns), to minimize the effect of onset orientations, DOX molecules are placed roughly 2 nm away from the carrier. As the simulation progresses (Fig. 2b, around 42 ns), some of the DOX molecules spontaneously move toward the carrier surface and form weak interactions with CN. It can be observed that one of the adsorbed drug molecules has interacted with the carrier via T-shape π–π stacking. In this configuration, the benzene ring of DOX is oriented perpendicular to the carrier surface. The other drug molecules interacted through H-bonds with the edge of the CN. According to the final snapshots (Fig. 2c), all the adsorbed DOXs are re-oriented during simulation time. Close inspection of these snapshots shows that the perpendicular molecule at the end of the simulation is changed to parallel orientation to the surface. Furthermore, the distance between this drug molecule and the carrier is reduced to eventually drug physiosorbed on the nanosheets. It is found that this drug, via its benzene rings, forms strong π–π stacking interactions with the CN where their vertical distances are about 0.2–0.3 nm. In this configuration, many interatomic interactions between DOX and CN are observed which preventing the leak of the drug during transportation. While the other molecules positioned on the CN edge are separated from the surface of the carrier at the end of the simulation (compare Fig. 2b,c). This finding can attribute to their weaker interactions with CN compared to the drug with parallel orientation. In general, it can be concluded that π–π interactions play a significant role in the adsorption of the DOX molecule on the CN surface. According to the obtained results, to enhance drug loading capacity, it suggested a CN in the bigger size can be used.

**Figure 3:**
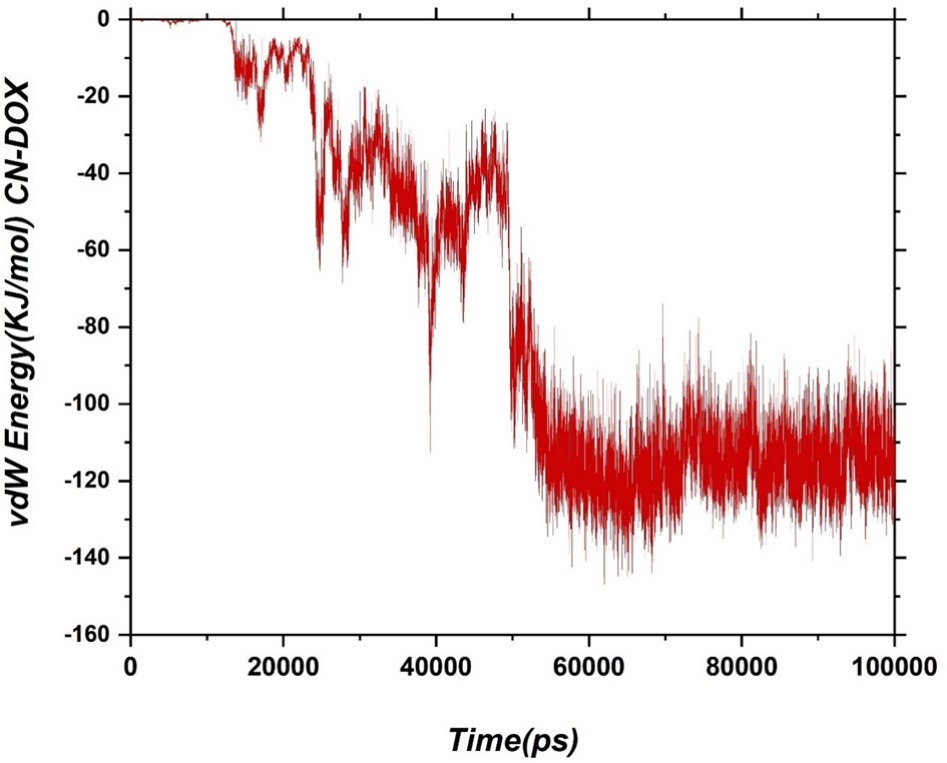
The interaction energy between the drug molecules and the carriers as a function of time.

Although a qualitative insight into the drug-carrier interaction is obtained from the snapshots, several analyses are employed to quantify the adsorption process. To interpret the adsorption process, the adsorption energies (vdW + Elec) are extracted from MD productions. The vdW interactions of the drug-carrier pair as a function of simulation time graphically are represented in Fig. 3.

**Figure 4:**
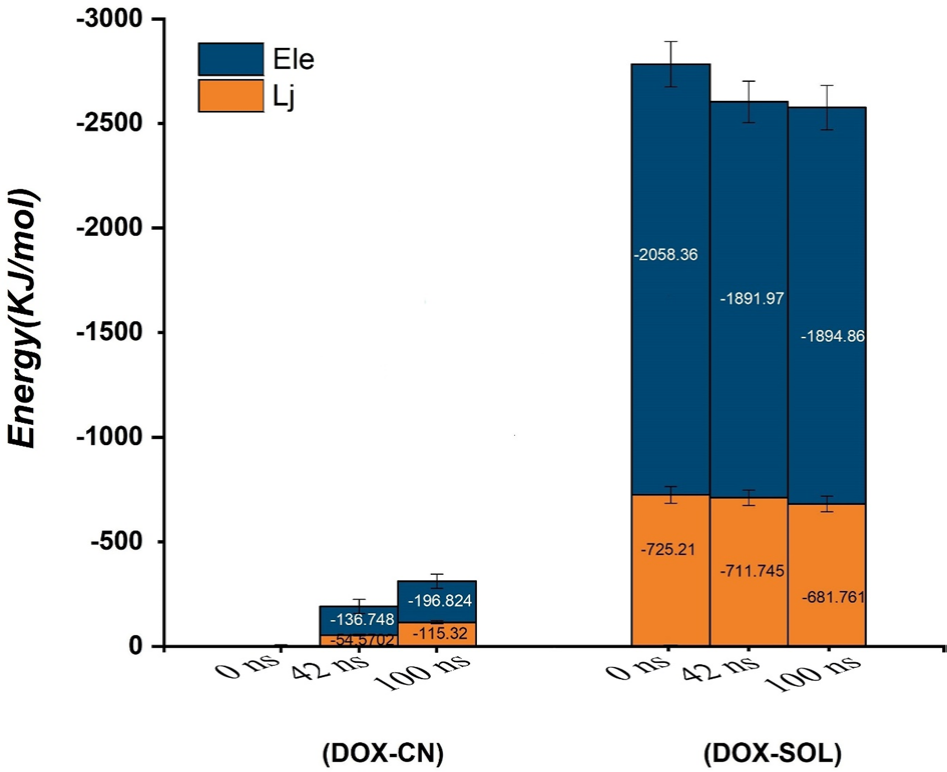
Average Van der Waals, electrostatic energies of the DOX-CN and DOX-SOL pairs at the three different times.

From 0 to 42 ns, the vdW energy has a decreasing trend, and due to drug re-orientations, its fluctuation is severe. Meanwhile, after 50 ns, by increasing interatomic interactions between DOX and CN surface, the vdW energy has reached the lowest value (≈ − 120 kJ/mol). This result means that DOX adsorption on the CN is thermodynamically stable, and π–π stacking is the main factor in drug stabilization. The average interaction energies and their standard deviations for the DOX-CN and water-DOX pairs at three different times are shown in Fig. 4.

In this Figure, the different energy contributions are also given separately. According to this Figure, since at 0 ns, the distance of DOX and CN is about 2 nm, both Elec and vdW energies for the DOX-CN pair are zero. The average vdW and Elec energies for DOX-CN interaction at time = 100 ns about 63% is more than those at time = 42 ns, which indicates that the DOX molecule tends to adsorb on the CN. Moreover, it is known that the adsorption tendency of DOX on the nanocarrier surface can be explored by comparison of its interactions with the CN and solvent. As can be seen in Figure, during the simulation, the interaction energy between the drug and the carrier is increased, while the amount of interaction energy between the drug and the solvent is decreased. These results reflect the fact that DOX can move spontaneously from the bulk toward the CN nanosheet surface.

**Figure 5:**
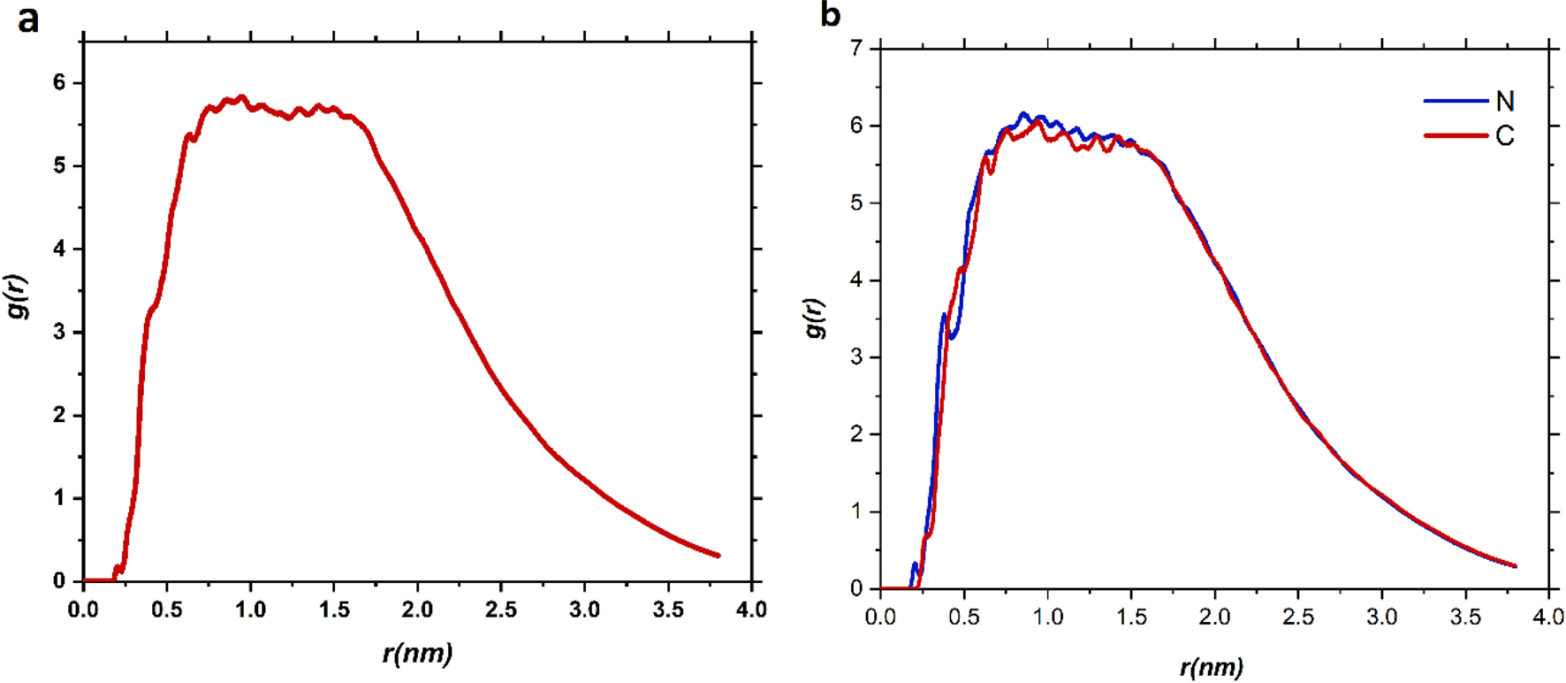
(**a**) Radial distribution function (RDF) of the centre of mass of the adsorbed drug molecule with respect to the carrier atoms. (**b**) atomic RDF profiles of N and C atoms with the adsorbed DOX molecule.

The radial distribution function (RDF), which is a weighted sum of intermolecular pair correlation functions^40^, describes the probability of finding the DOX molecule at a certain radial distance from the CN surface. The respective plot for averaged RDF during the MD production against the radial distance is given in Fig. 5.

According to the Fig. 5a, the main peak of g(r) locates at r = 0.96 nm for the DOX molecule. This peak can be attributed to the formation of HB and vdW interactions between the DOX and carrier. These observations have a good correlation with the snapshots previously discussed.

In previous studies, it is found that the interaction of host molecules with N moieties of CN is stronger than C moieties^41,42^. To investigate such behaviour in the present study, the atomic RDF of N and C atoms with the adsorbed DOX molecule is calculated and given in Fig. 5b. The location and intensity of RDF peaks for both atoms are the same, which shows that the contribution of N and C atoms in interaction with the drug is the same. The observation can be related to the big size of the drug and also the formation of $\pi -\pi$ stacking interaction between DOX and CN surface.

**Figure 6:**
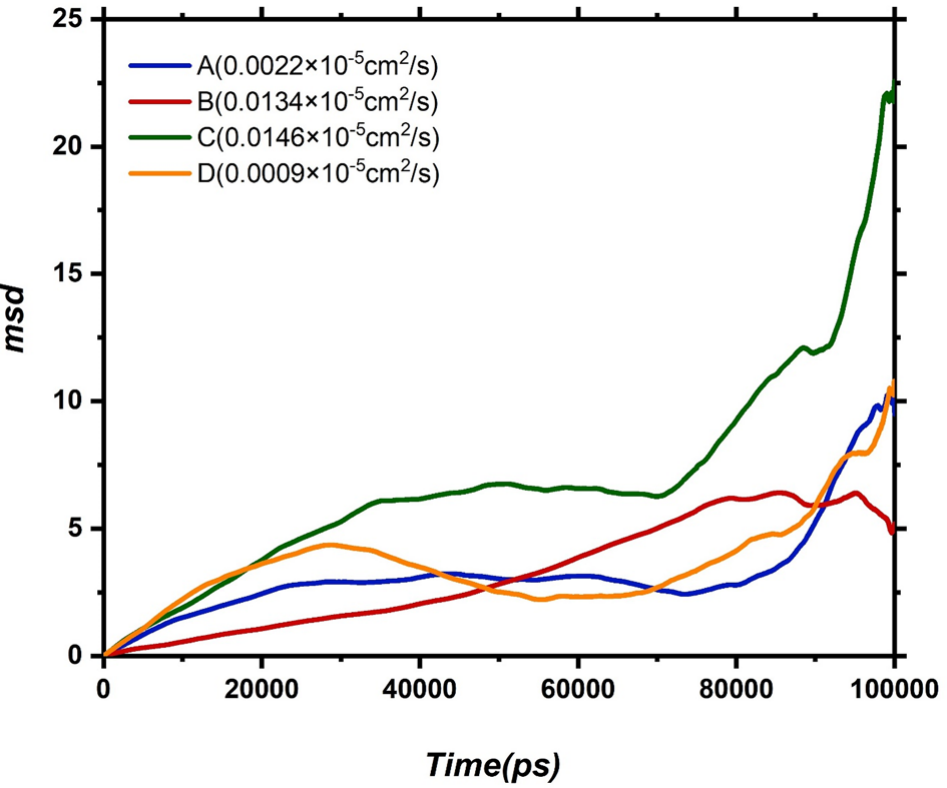
Mean square displacement (MSD) of drug molecules versus simulation time with corresponding D values in the investigated systems. (**A**,**D**) two adsorbed drugs to the edge of the carrier, (**B**) adsorbed drug in parallel on the carrier, (**C**) unabsorbed drug**.**

The dynamics of drugs are investigated both in terms of orientational and translational mobility. Translational dynamics are analyzed through the self-diffusion coefficient (D), which is derived from the mean squared displacement (MSD) using the Einstein relation^43^:

2 $${lim}_{t\to \infty }<{\left|\left|{r}_{i}\left(t\right)-{r}_{i}\left(0\right)\right|\right|}^{2}>=6Dt$$

where ri(0) and ri(t) are the positions of the particle at time 0 and t, respectively. The term in the bracket < … > represents the MSD of the drug. Figure 6 shows values of D and MSD plot for all drugs as a function of simulation time.

For the drug with parallel orientation, MSD initially exhibits a linear increase with a slope around 8 against time which after adsorption becomes nonlinear. This indicates that the DOX molecules follow normal Einstein dynamics far from the CN surface while following Rouse mode close to the CN surface. As it is obvious from Fig. 6, the diffusion coefficient for the drug which has the highest interaction with CN, is lower than the other drug molecules. This result can be explained by the fact that adsorption of DOX on the CN surface restricts the drug movement. Interestingly, for the adsorbed drugs on edge, the MSD plot initially has an increasing trend, and after adsorption on the CN edge, their mobility decreased. Finally, after desorption of DOX molecules from the CN, MSD again increased.

### Drug delivery system close to membrane cells:

**Figure 7:**
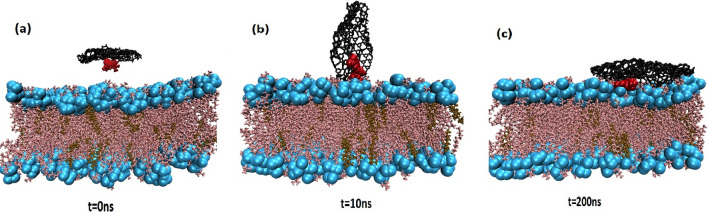
Evolution of the progressive insertion of CN-DOX into the lipid bilayer at three times of the simulation. (**a**) 0 ns, (**b**) 10 ns, (**c**) 200 ns.

In order to understand the interaction mechanism of DDS with the lipid bilayer membrane, a simulation system is designed. One typical representation for interactions of DDS with the POPC lipid bilayer at various time throughout the simulation is illustrated in Fig. 7.

Initially, the CN-DOX complex is placed in parallel orientation at a distance of 3 nm above the membrane (Fig. 7a, at 0 ns). During the simulation, the DDS spontaneously moves from the bulk toward the membrane. It is observed that in ~ 10 ns, the orientation of the DDS complex change and prefer vertical orientation respect to the membrane (Fig. 7b, at 10 ns). It is also found that this system remained in this configuration until ~ 107 ns. This behaviour can be related to the competition of water and POPC in interaction with DDS. Finally, at 107 ns, as shown in Fig. 7c, the DDS is adsorbed on the surface of the lipid membrane and stayed therein for the rest of the simulation time. According to these snapshots, it can be found that the polar heads of the membrane play an important role in interacting with the DDS.

**Figure 8:**
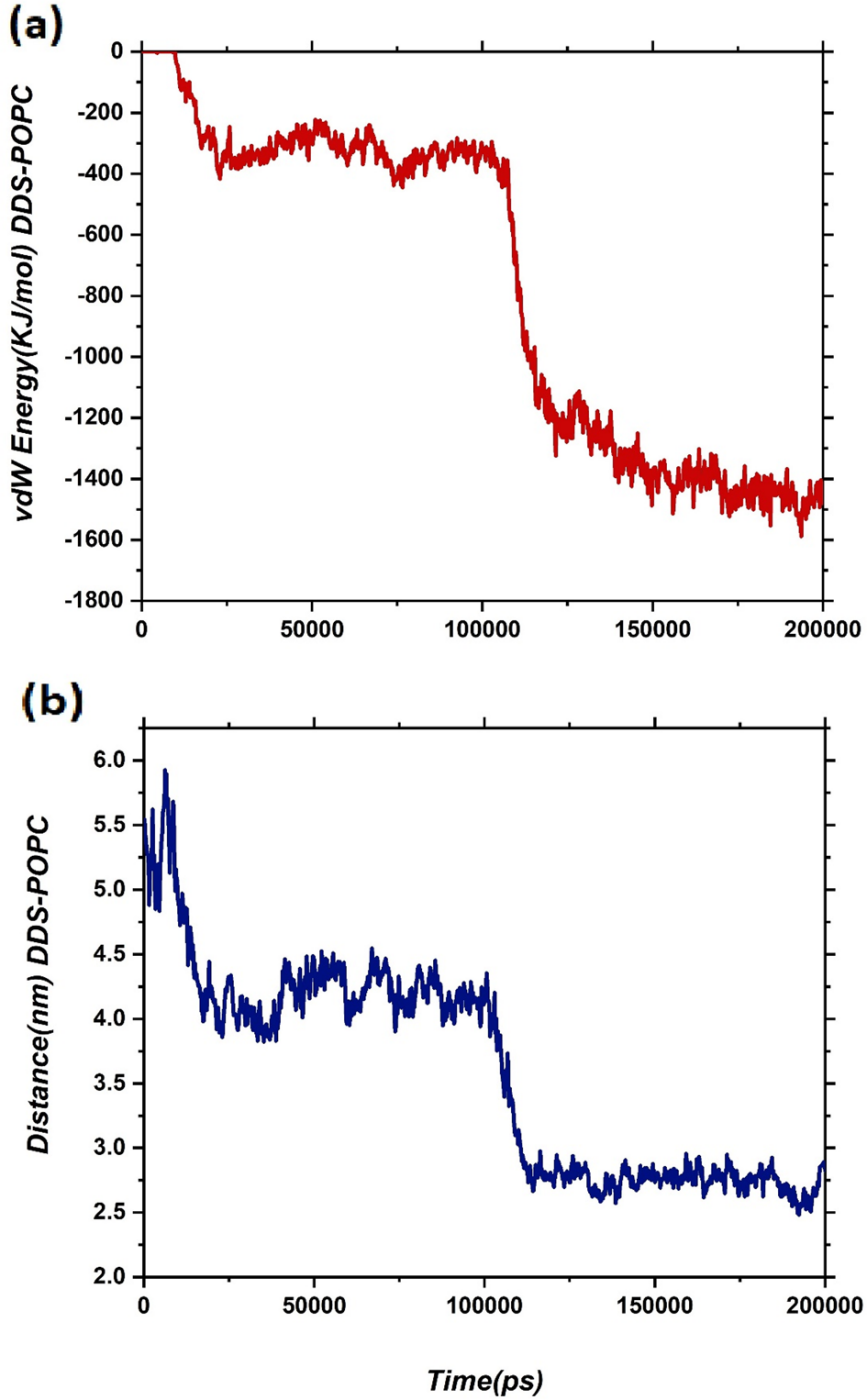
(**a**) Van der Waals energy and (**b**) COM distance between CN-DOX complex and lipid bilayer as functions of simulation time.

In order to inspect the interaction of DDs with the membrane, the vdW energy and the centre of mass (COM) distance between the membrane and the CN-DOX complex as a function of the simulation time are calculated and given in Fig. 8.

It should be noted that this energy is divided into two parts because the DDS includes DOX and CN nanosheet which each of them can interact with the bilayer. The difference of interaction energy between the CN-DOX complex and the membrane at the beginning and end of the simulation time is reached ≈ − 1479 kJ/mol (the contribution of CN and DOX is about 1370 and 130 kJ/mol, respectively). This finding indicated that the DDS complex strongly interacts with the membrane and rapidly adsorbed into it. Furthermore, the COM diagram shows a similar behaviour with the vdW energy diagram, which confirms our conclusion (see Fig. 8b). As can be seen in Fig. 8b, the COM distance between the CN-DOX and the lipid bilayer at two times of about 10 and 107 ns is gradually reduced. The Vdw and Ele energies between different components of the studied system are calculated and reported in Table 1.

**Table 1:** Average electrostatic (Ele) and van der Waals (Lj) interaction energies between the different components of the investigated system.

Pair	Energy	Time	
		0 ns	200 ns
Complex-POPC	Lj	0	− 1479.56
	Ele	0	− 3164.22
Complex-SOL	Lj	− 2823.66	− 2148.96
	Ele	− 12,170.12	− 7771.30
CN-DOX	Lj	− 152.57	− 155.39
	Ele	− 46.19	− 45.60

It is observed that the Ele energy has a significant contribution to the adsorption of DDS into the cell membrane, which can be due to the strong interaction of POPC head groups with the CN-DOX complex. Therefore, the Ele interaction part has an important effect on the stability of the DDS on the lipid bilayer. Moreover, Table 1 clearly shows that by moving the DDS from bulk toward the lipid bilayer, the contribution of DDS interaction energy with solvent is considerably reduced. It is worth noting that the amount of this energy loss with water is almost compensated by the increase in interaction energy with the membrane. Furthermore, visual inspection of the MD production indicated that during simulation time, any passive diffusion or release of the drug molecule does not occur. Close inspection of Table 1 shows that the interaction energy of DOX and CN during the simulation time is constant. It is confirmed that adsorbed DOX molecules on the CN surface can diffuse into the membrane cell. While the penetration of free DOX into the spheroids is limited, which caused to reduces its activity against centrally located cells^44,45^. Also, Hashemzadeh et al.^46^ were reported that DOX in complex with graphene could not diffuse into the lipid bilayer. Therefore, it can be concluded that the CN nanosheet is suitable for the delivery and stabilization of drugs at the membrane surface.

**Figure 9:**
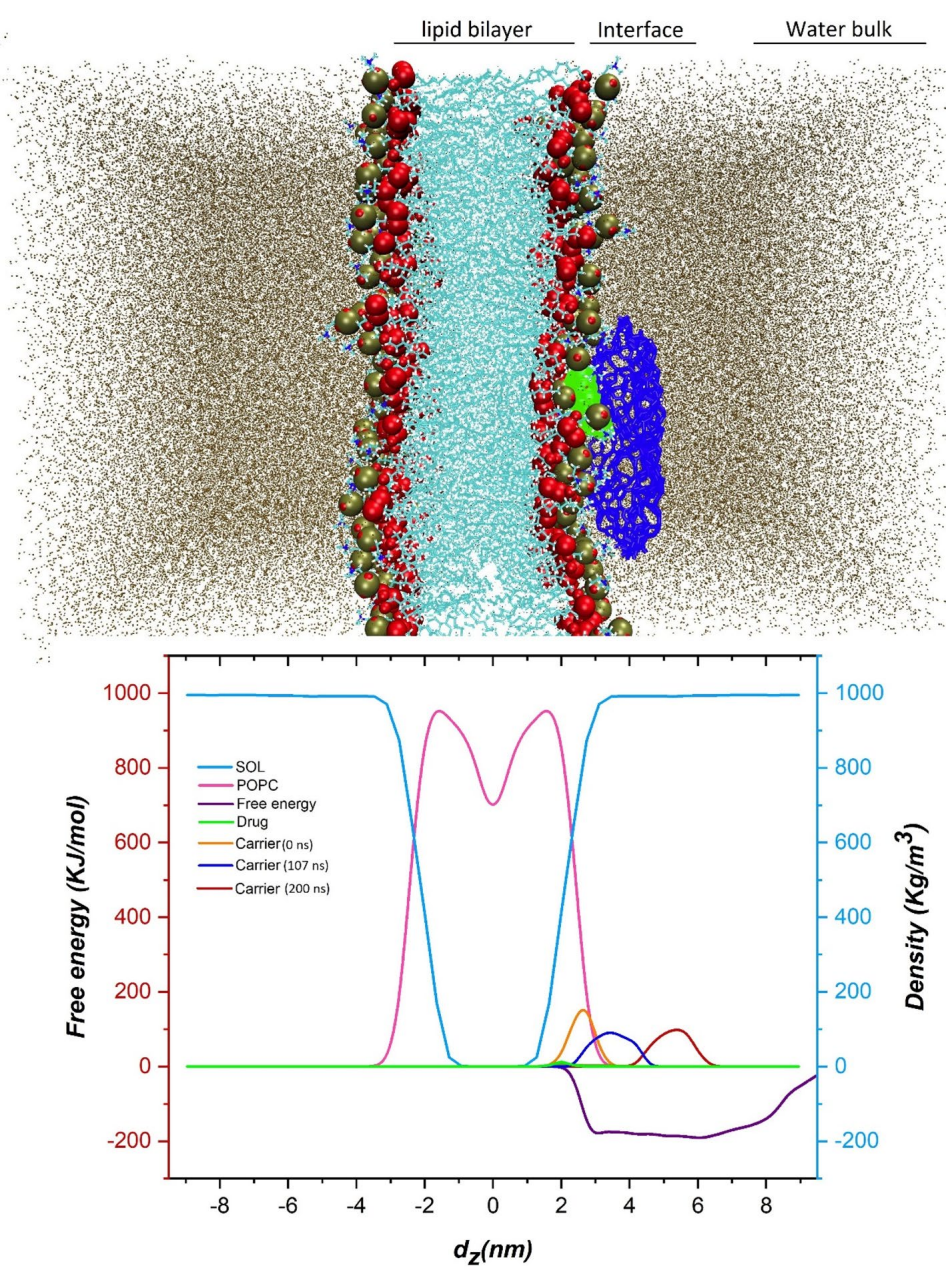
Free energy profile for penetration of DDS into the membrane cell and density profiles of water, POPC, carrier in different time points(0 ns, 107 ns, and 200 ns), and drug. Both free energy and density are plotted as a function of the collective variable CV, i.e. the fractional coordinate along the direction (z) normal to the membrane.

Free energy calculations can be employed to study various biological systems as effective methods in theoretical chemistry^47,48^. Several methods have been introduced to enhance the sampling of biological processes^24,25^. Metadynamics is one of the prospering techniques among free energy calculation methods for exploring the energy of long-time processes^38^. Here, metadynamics simulation is performed to achieve the free energy surface as a function of the collective variable D, which is defined as the distance between DDS and the membrane. FES along the direction (z) by using the sum_hills program is obtained and depicted in Fig. 9.

**Figure 10:**
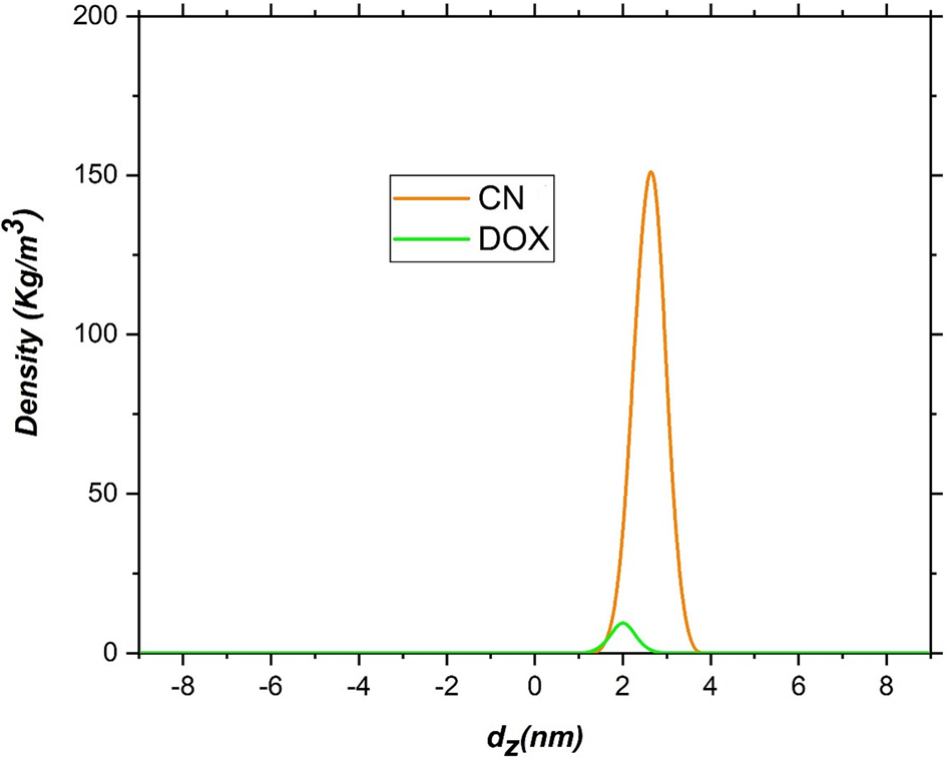
The density profile of DOX and CN in the difuusion simulation box.

To better understand the arrangement of the various components in the system under consideration, the density profiles along the z-direction alongside free energy has been provided in Fig. 9. It should be noted that to calculate the density, the "gmx density" module has been used and the POPC membrane is selected as the reference. Examination of the density profile is clearly showing that during 200 ns, the bilayer membrane is properly equilibrated. In addition, the analysis of the density profile confirms that at the start of the simulation, the DOX-CN complex is positioned out of the membrane at a distance about of 3 nm. Besides, it is observed that this complex moves rapidly from the water bulk region to the membrane surface without destroying the hydration layer. This obtained result may be due to the high tendency of the DDS to adsorb on the POPC surface. In 107 ns, the density profiles of the membrane and the DDS overlap together, indicating contacts between the complex and the lipid headgroups are beginning (see Fig. 9). It is found that the overlap between the density profile of the drug and the nanosheet remain to the end of the simulation time (Fig. 10). These obtained results are nicely confirmed by the energy results and the provided snapshots shown in Fig. 7. The FES has also been used to more quantitatively illustrate the transposition of the DDS at different distances from the lipid membrane. The energy value by moving the complex toward the interface is sharply decreased, but when it reaches the interface, the decrease in energy occurs with a moderate slope. This trend continues until the free energy reaches the minimum state inside the polar head of the membrane. In other words, the minimum point (with energy about − 200 kJ/mol) of FES is located at a distance of about 1.4 nm from the membrane centre of mass. It is worth noting that to reach this point, the drug delivery system does not face any significant energy barrier. These observations illustrate that the carrier-drug complex has a high tendency to adsorb on the membrane cell.

**Figure 11:**
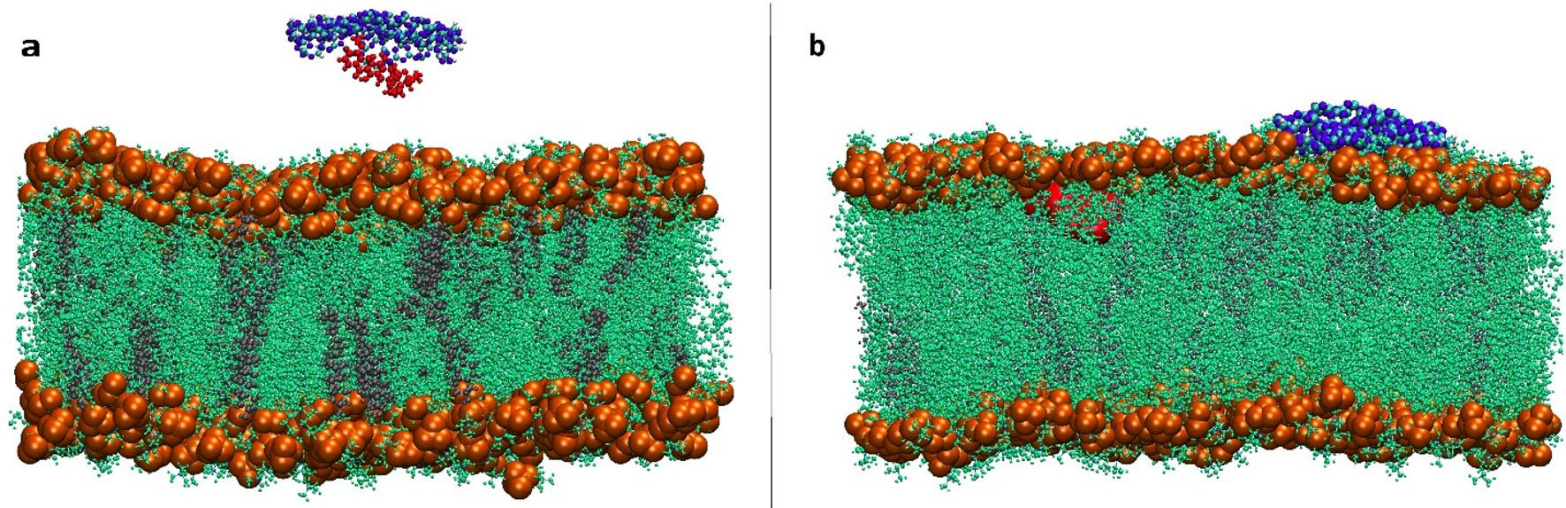
Evolution of the progressive insertion of CN-DOX into the lipid bilayer at two times of the simulation. (**a**) 0 ns, (**b**) 100 ns.

To study the effect of CN size on the DDS diffusion mechanism, another system is designed. In this system, a smaller CN, including 168 Carbon, 217 Nitrogen, and 24 Hydrogen, as the carrier is selected, and one DOX adsorbed on it. The diffusion mechanism of this DDS in the presence of the lipide bilayer is investigated. In Fig. 11, the initial and final snapshots of the designed system are depicted.

As can be seen in this Figure, similar to the DDS based on the bigger CN, the small CN nanosheet spontaneously move towards the membrane cell. However, during the diffusion DOX molecule is desorbed from the CN surface and separately interacts with lipide bilayers. This behaviour can be related to the weaker interaction of DOX with the smaller CN nanosheet that caused to desorption of the drug from the carrier surface. The energy values for the interaction of the small CN with POPC are listed in Table 2.

**Table 2:** Average electrostatic (Ele) and van der Waals (Lj) interaction energies between the different components of the investigated system.

Pair	Energy	Time	
		**0 ns**	**100 ns**
Complex-POPC	Lj	0	− 803.07
	Ele	0	− 1802.32
Complex-SOL	Lj	− 4655.22	− 1062.31
	Ele	− 7053.95	− 1455.95
CN-DOX	Lj	− 51.63	0
	Ele	− 72.99	0

The comparison of the interaction energy terms in the presence of big and small CNs indicated that the interaction is more in the larger case and consequently has a greater tendency to penetrate the membrane.

## Conclusion

In this study, by means of MD simulation, we investigate the interaction of CN nanosheet with DOX anticancer drug. In the CN-DOX system, the drug molecule prefers parallel orientation with respect to the carrier surface and forms HB and π-π interactions. This conclusion is confirmed by the analysis of adsorption energy, which indicates both van der Waals and electrostatic energy contributions between DOX and CN are increased. Furthermore, in the present study, the transfer process of the loaded drug on the carbon nitride nanosheet to the POPC membrane surface is investigated. It is found that the CN-DOX complex could rapidly adsorb into the membrane, which is driven mainly by the electrostatic interactions between polar groups of the membrane and the CN-DOX complex. Furthermore, the overlap between the density profile of the drug and the nanosheet exists up to the end of the simulation time. Moreover, to explore the free energy surface of the studied system, metadynamics simulation is employed. It is observed that when the complex approaches the surface of the membrane, its free energy is decreased. In the global minimum, the DOX-CN complex is located among polar groups of the bilayer. In summary, the global minimum in the free energy surface is about − 200 kJ/mol. In this way, it can be concluded that the choice of a CN sheet as a nano vector paves the way for DOX drug delivery to the membrane surface. We believe that this work has potential applications in medicine.

## Data Availability

The input and output of simulation systems are available from the at EMBL-EBI (https://www.ebi.ac.uk/biomodels) and can be accessed with the identifier MODEL2102240001.
